# Plant root carbon inputs drive methane production in tropical peatlands

**DOI:** 10.1038/s41598-025-87467-w

**Published:** 2025-01-25

**Authors:** N. T. Girkin, A. Siegenthaler, O. Lopez, A. Stott, N. Ostle, V. Gauci, S. Sjögersten

**Affiliations:** 1 School of Biosciences, University of Nottingham, Sutton Bonington, LE12 5RD UK; 2 School of Environment, Earth and Ecosystem Sciences, The Open University, Milton Keynes, MK7 6AA UK; 3 Smithsonian Tropical Research Institute, Tupper Building (401), Balboa, Ancón, Panama; 4 Inter-American Institute for Global Change Research, Edificio 104, Ciudad del Saber, Clayton, Ancón, Panama; 5 Centre for Ecology and Hydrology, Lancaster Environment Centre, Lancaster, LA1 4AP UK; 6 Lancaster Environment Centre, Lancaster University, Lancaster, LA1 4YQ UK; 7 Birmingham Institute of Forest Research (BIFoR), School of Geography, Earth and Environmental Sciences, University of Birmingham, Birmingham, B15 2TT UK; 8 School of Geography, Earth and Environmental Sciences, University of Birmingham, Birmingham, B15 2TT UK

**Keywords:** Tropical peat, Methane, Stable isotope labelling, Girdling, PLFA, Biogeochemistry, Environmental sciences

## Abstract

Tropical peatlands are carbon-dense ecosystems that are significant sources of atmospheric methane (CH_4_). Recent work has demonstrated the importance of trees as an emission pathway for CH_4_ from the peat to the atmosphere. However, there remain questions over the processes of CH_4_ production in these systems and how they relate to substrate supply. Principally, these questions relate to the relative contribution of recent photosynthetically fixed carbon, released as root exudates, versus carbon substrate supply from the slowly decomposing peat matrix to CH_4_ emissions within these ecosystems. Here, we examined the role of root inputs in regulating CH_4_ production inferred from soil emissions using a combination of in situ tree girdling, in situ^13^C natural abundance labelling via stem injections, and a ^13^CO_2_ labelling of transplanted plants of two contrasting plant functional types, a broadleaved evergreen tree, and a canopy palm. Girdling of broadleaved evergreen trees reduced CH_4_ fluxes by up to 67%. Stem injections of trees and palms with a natural abundance label resulted in significant isotopic enrichment of CH_4_ fluxes, reinforcing the link between root carbon inputs and peat CH_4_ fluxes. *Ex situ*^13^CO_2_ labelling of plants resulted in significant ^13^C enrichment of peat CH_4_ fluxes. Taken together, our results demonstrate for the first time that plant root exudates make a substantial contribution to CH_4_ production in tropical peatlands.

**Supplementary Information:**

The online version contains supplementary material available at 10.1038/s41598-025-87467-w.

## Introduction

Tropical peatlands are a globally important carbon stock, are a significant contributor to global wetland methane (CH_4_) emissions, and highly vulnerable to climate change^1–4^. The net balance of CH_4_ fluxes from tropical peatlands is controlled by the water table position, with waterlogged anoxic conditions a prerequisite for methanogenesis, while methanotrophy dominates under drier aerobic conditions. Vegetation is also a key regulator of emissions, determining initial peat properties^5^, providing a regular litter supply^6^, as well as a mechanism of gas transportation^7^. However, the regulatory role of roots, which can release significant quantities labile carbon^8,9^ and oxygen^10^ at depth, is still unclear.

Significant diurnal variation in CO_2_ fluxes from tropical peatlands in Indonesia and Panama have previously been reported, with fluxes increasing rapidly throughout daylight hours and declining overnight^11,12^. Positive correlations have been found between diurnal changes in CO_2_ efflux and air temperature, possibly due to changes in decomposition of organic matter^11^, but also with rates of photosynthesis^13^ indicating the important contribution of root exudates to the labile peat carbon pool, and microbial respiration^8,9^. Similarly, there is evidence for diurnal variation in CH_4_ fluxes driven by changing rates of photosynthesis in wetland plants^14–16^. The link between CO_2_ and CH_4_ fluxes and recent photosynthetically fixed carbon is likely to be species specific through a combination of differences in root exudate profiles^17^, oxygen inputs^10^, rooting structures^12^, and contrasts in microbial community abundance and function^18,19^. Moreover, trees are also known to be an important pathway for CH_4_ transport, accounting for between one and two thirds of ecosystem fluxes^7,20^.

Techniques developed for partitioning the drivers of soil CO_2_ dynamics, for example trenching, severing roots mechanically, girdling (the removal of bark around the stem to reduce the flow of photoassimilates to the roots and mycorrhizae), and stable isotope labelling with ^13^C enriched and natural abundance isotopes^21^ also can be applied for interpreting CH_4_ dynamics^40^. Stable isotope labelling has been widely used to assess the contribution of recent photosynthetically fixed carbon to net CO_2_ fluxes in a range of systems including agriculture and plantation crop systems^22,23^, forests^24,25^, grasslands^26,27^, temperate peatlands^28,29^, and rice paddy fields^15,30^. Transfer of carbon to soil microbial communities can occur within hours but is dependent on plant species and size^25,31,32^. While many studies introduce a ^13^CO_2_ label in the gaseous phase for photoassimilation, stem injection of a label negates the challenges of labelling full sized trees in situ^32^. In addition, stable isotope labelling, particularly using highly enriched labels, can have significant associated costs making it often a prohibitively expensive way to demonstrate carbon flow pathways at scale. In contrast, natural abundance labelling, in exploiting differences in isotope ratios between sugars produced by C3 and C4 plant metabolism offers a cost-effective approach for labelling in situ.

Stable isotope labelling also allows assessment of key microbial groups driving the use of recent photoassimilates relative to older organic carbon. Within organic grassland soils and boreal peats, fungal biomarkers have frequently been shown to be rapidly enriched following labelling, with a decreasing ^13^C enrichment over time, indicating the ability of fungi to rapidly incorporate plant derived carbon^22,26,33^. Significant enrichment over short timescales following labelling has also been demonstrated for Gram negative bacteria in agricultural soils^34^, and peats^22^. Rhizosphere soils have also been found to have greater abundance of Gram negative bacteria than bulk soils implying a further possible role in decomposition of labile carbon derived from recent plant inputs^35,36^. In the longer term, enrichment of Gram positive bacteria suggests an important role in the degradation of more recalcitrant organic matter^22,37^. Contrasts in the relative incorporation between PLFA groups between different plant communities would therefore likely indicate functional differences in the microbial communities under contrasting peat types.

In this study, we examined the role of trees in the CH_4_ production process. assess the role of root inputs of carbon using three methods: (i) in situ girdling of tree stems; (ii) in situ stem injection of a natural abundance label; (iii) ex situ^13^CO_2_ labelling of plants. We hypothesised: (i) girdling would significantly reduce peat surface CH_4_ fluxes due to reduced root inputs; (ii) stem injections of a natural abundance label and ^13^CO_2_ labelling would significantly enrich CH_4_ fluxes following labelling; (iii) CH_4_ enrichment of peat surface emissions following labelling will differ between plant species.

## Methods

### Field site, vegetation and peat properties

To quantify the role of roots and root inputs of carbon in driving tropical peatland CH_4_ dynamics, we integrated an in situ girdling (conducted in September–October 2013), a natural abundance labelling (conducted February 2015) and an ex situ^13^CO_2_ pulse-labelling pot experiment (February–May 2016). All studies were conducted at the San San Pond Sak freshwater and marine wetland located in Bocas del Toro province, Panama, under ANAM research/collection permits SE/P-29-13 and SE/P-34-13. The wetland features an 80 km^2^, 8 m deep ombrotrophic peatland at Changuinola initiated 4,000–5,000 years ago^38^. Coastal vegetation is dominated by *Rhizophora mangle*, followed by *Raphia taedigera* palms, mixed species forest stands, *Campnosperma panamensis* broadleaved evergreen tree stands and *Myrica-Cyrilla* bog-plain. Accompanying the succession of plant communities is a strong decline in nutrient availability towards the interior of the wetland^39^, alongside similar gradients in organic matter properties^5^ and microbial community structure^40^.

Between 2002 and 2016 mean annual air temperature was 25.7 °C, with little intra-annual variability. During the period of sampling mean temperature was 26.9 °C. Over the same period, mean annual rainfall was 3,293 mm, with a mean of 173 mm in February to May 2016. Mean sub-surface peat temperature is 25.0 °C. At the study sites, the water table fluctuates from just above to just below the peat surface, with a range of approximately 20 cm^41^.

### Tree girdling

To determine the role of transfer of recently photosynthesied carbohydrates released in to the rhizosphere for CH_4_ emissions from the peat surface we carried out a girdling experiment in September 2013. Girdling involved removal of the bark, xylem and phloem tissue around the tree trunk to stop transfer of carbohydrates to areas below the point of girdling (Hogberg et al., 2002). We carried out a girdling experiment in September to October 2013 in a monodominant *C. panamensis* stand within the San San Pond Sak peatland. Paired *C. panamensis* trees (*n* = 8) were randomly allocated a treatment (control or girdled). Diameter at breast height ranged between 50 and 150 cm with a mean diameter of 85 cm. Girdling was carried out beginning of October by removing 2–3 cm of tissue around the entire tree.

Soil CH_4_ flux was monitored before, immediately after and then again after two weeks at the same point, measured 1 m from the trunk of each tree in a randomly selected direction, to account for the previously reported spatial heterogeneity in rooting zone CH_4_ dynamics^40^. The mean water table depth across the site was 0.75 cm above the surface of the peat at the time of sampling, in keeping with the previously reported range of values for the site^41^.

For measuring CH_4_ fluxes, we inserted a lightweight polypropylene (PP) rim (inner diameter: 30 cm; height: 15 cm) 10 cm deep into the peat surface, the day prior to measurement. For the measurment, we then slotted a cylindrical chamber (diameter 30 cm, height 20 cm) into the the rim ensuring an air-tight seal. To reduce disturbance of the soil surface a 120 × 50 cm polystyrene board was used to kneel on during sampling. It is plausible that accessing the sampling locations resulted in ebullition. The chambers were connected to an Ultra-Portable Greenhouse Gas Analyser (UGGA, Los Gatos Research Inc., Mountain View, USA) via two 4.6 m long and 5 mm inside diameter polytetrafluoroethylene coated polyvinyl chloride parallel tubes (Nalgene, Rochester, USA) set in a continuous flow mode operating as a closed loop with a flow of 2–4 L min^−1^. The UGGA measured CH_4_ with the Off-Axis Integrated Cavity Output Spectroscopy (OA-ICOS) at a frequency of 0.33 Hz. Gas concentrations were then measured for 5 min. The analyser’s uncertainty in the range of 0.01 ppmv to 100 ppmv of methane is < 1% without calibration and the precision is ± 0.6 ppb over a period of 100 s.

The rates were calculated from linear regressions made between the concentration changes starting after an equillibration period of 90 s and the elapsed runtime. After accounting for the chamber volume, which varied between measurements depending on the requiered chamber size (the range of chamber size was 0.28 to 1.49 dm^3^ and 95 to 715 cm^2^ for the volume and the area, respectively), rates were then expressed relative to the exchange surface area. All flux series was inspected to ensure ebullition was not affecting the calculated fluxes.

The ambient fluxes were corrected to reference fluxes using the following transformation:

1 $${{\text{F}}_{{\text{ref}}}}={\text{ }}{{\text{F}}_{{\text{amb}}}}\left[ {{{\text{P}}_{{\text{amb}}}}/{{\text{P}}_{{\text{ref}}}}} \right]*\left[ {{{\text{T}}_{{\text{ref}}}}/{{\text{T}}_{{\text{amb}}}}} \right]$$

where F_ref_ = flux corrected to reference conditions, F_amb_ = flux measured at ambient conditions, P_amb_ = atmospheric pressure at ambient conditions, P_ref_ = pressure at reference conditions (1 atm), T_ref_ = temperature at reference conditions (298 K), T_amb_ = temperature at ambient conditions in K. During subsequent data analysis, three pairs with negative CH_4_ fluxes i.e. CH_4_ was oxidised, were discounted as CH_4_ oxidation would mask the contribution of roots exudates to fluxes^10^.

### Natural abundance labelling

Six *C. panamensis* trees and six *R. taedigera* palms were selected for stem injection of a C4 derived sugar based on similar heights and diameter-at-breast-height (DBH). A 2 cm hole was drilled at a 45° angle into trees at approximately 30 cm above the peat surface. A 30 cm section of rubber tubing was silicon sealed in place using a non-emitting sealant. Three trees of each species were randomly selected for labelling. For each plant, 100 g of C4 derived sugar (− 12.11 ± 0.009) was dissolved in 2 L of deionised water and connected to the tubing through a hole in the lid. The bottle was subsequently inverted to allow the flow of the solution into the stem. Unlabelled controls received 2 L of deionised water. Peat surface CH_4_ fluxes were subsequently measured using the closed chamber technique as above, but with duplicate samples collected after 20 min for ^13^C analysis. Fluxes were measured immediately prior to labelling and 1, 4, 5 and 7 days following labelling. At the conclusion of the study, bottles from which only a limited volume of water had been lost (one labelled and one control *C. panamensis* trees, and one labelled and two control *R. taedigera* palms) were discounted from subsequent analysis. Due to a lack of statistical replication for *R. taedigera* palms, data from individuals of both species was combined (*n* = 4 for treatment; *n* = 3 for control).

### ^13^CO_2_ pulse labelling assay

Plant species were selected based on their high relative abundances within the forest stands at Changuinola. Peats derived from *C. panamensis* and *R. taedigera* have previously been shown to differ significantly in terms of in situ GHG production and organic matter properties^5,42^ and microbial community structure^43^. In addition to *C. panamensis* and *R. taedigera*, which form monodominant forest stands, *Symphonia globulifera*, a second broadleaved evergreen tree was also selected for labelling. During transplantation, there was high mortality of *C. panamensis* saplings, necessitating the selection of an additional plant species. *S. globulifera* has a tall trunk supported by buttress roots, with lenticels for root oxygenation giving it a similar physiology to *C. panamensis* which also has lenticels.

Nine *R. taedigera*, *C. panamensis* and *S. globulifera*, selected based on similar height (30–40 cm) and diameter-at-breast-height (DBH, 0.3–0.4 cm), were collected from the mixed forest stand. Peat around the plants was removed to a 25 cm depth and in a 20 cm radius around plant stems to ensure removal of the intact root system. Plants were placed in pots and transferred to the Smithsonian Tropical Research Institute research station on Isla Colón. Plants were maintained at the research station for three months prior to the beginning of labelling, including regularly watering to maintain the water table at 1 cm above the peat surface, and placing in partial shade to mimic in situ conditions.

Labelling was conducted using custom made Perspex chambers (15.71 L) fitted with a suba seal for labelling and sampling and with a battery powered fan (Evercool EC4010M12EA) powered by 9 V batteries to ensure mixing of headspace gases. Labelling was conducted between 9 am and 5 pm in direct sunlight to maximise plant photosynthesis rates. Six plants from each species were randomly selected for labelling, with three of each species retained as unlabelled natural abundance controls. The Perspex chamber was placed over the selected saplings and gently pushed into the peat to ensure a tight fit. Plants were labelled with ^13^CO_2_ (99 atom % ^13^C; Cambridge Isotope Laboratories) added in pulses of 100 mL. Chambers remained in place for 40 min to allow prolonged uptake of the label before lifting to allow the plants cool and condensation to dissipate. This time was chosen, as during trials, temperatures in the headspaces gradually rose over time, in some instances reaching over 40 °C. Labelling was repeated for a total of five pulses during the course of the day, beginning on 18th May 2016. This approach does incur the risk of directly labelling the methanogenic communities, thereby driving hydrogentrophic methanogenesis. However, the elevated water tables used in our experiment will have acted in part as a barrier to diffusion for atmospheric CO_2_, as they do for oxygen, thereby resulting in the anoxic conditions beneath the peat surface that are required for methanogenesis. Our previous work shows relatively high dissolved oxygen in surface waters that will result in the dominance of methanotrophy^10^.

Smaller headspaces (0.37 L) were used for sampling air directly from the peat surface for later CH_4_ and ^13^CH_4_ analysis. Duplicate headspace samples were collected one day prior to labelling, and one, three, seven and 14 days post labelling. Samples (20 mL) were collected immediately following the fitting of the headspace and after 10 and 20 min, and injected into 12 mL pre-evacuated glass exetainers fitted with a screw cap septum.

At the conclusion of the pot experiment four *C. panamensis*, four *S. globulifera* plants and one *R. taedigera* palm were dead and samples collected from these pots were excluded from further analysis. However, it is unclear if the high mortality was driven by treatment as there was high plant loss during transplantation from the site. Samples from *C. panamensis* and *S. globulifera* were thus combined due to their broadly similar physiologies to assess the response of broadleaved evergreen trees for the purposes of statistical analysis. Both species feature similar rooting structures and were of similar height, DBH and biomass with no significant differences in associated peat properties. This combination of three species is subsequently referred to as plant type, on the basis of the different physiologies and morphologies between broadleaved evergreen trees (C. *panamensis* and *S. globulifera*), and the palm (*R. taedigera*).

### CH4 and 13CH4 isotopic analyses

CH_4_ concentrations were quantified using gas chromatography (GC) using a single injection system fitted with a 1 mL sample loop, using H_2_ as a carrier gas and a non-polar methyl silicone capillary column (GC-2014; Shimadzu, Milton Keynes, UK). CH_4_ was detected using a flame ionization detector. Samples that were under-pressurised at the time of analysis were discarded; these duplicate samples were not subsequently analysed by GC-C-IRMS.

For determination of δ^13^C-CH_4_, headspace gases were manually injected into an Isoprime Trace gas analyser, which was coupled to an Isoprime continuous flow isotope ratio mass spectrometer (Elementar UK, Stockport). Samples were initially passed through a Magnesium perchlorate/ Carbosorb scrubber trap at a flow rate of 20 mL min^−1^ to remove water and CO_2_. CH_4_ was then oxidised in a combustion furnace using a braided platinum/copper/nichrome furnace wire inside a ceramic furnace tube (200 mm × 0.4 mm i.d.) heated in a furnace to 960 °C. A preparation flow rate of 10 psi was required to give a flow rate of 20 mL min^−1^ through the furnace at full operating temperature. Calbiration was achieved using CH_4_ standards cross calibrated with a CO_2_ reference gas, calibrated against NIST REF-Heavy Palaeomarine Origin (CO_2_) (RM 8562) and NIST REF-Biogenic Modern Biomass Origin (CO_2_) (RM 8564). δ^13^C-CH_4_ was expressed in per mil (‰). The reproducibility of δ^13^C-CH_4_ was better than ± 0.2‰.

### PLFA and 13 C-PLFA analysis

PLFAs were extracted from peat collected 14 days following labelling following the Bligh and Dyer protocol (1959). Total lipids were extracted from 500 mg of freeze-dried peats using a citrate buffer (0.15 M, pH 4), 1.9 mL chloroform (CHCl_3_), 3.8 mL methanol (MeOH), and 2 mL of Bligh and Dyer reagent (prepared at a 1: 2: 0.8 volume ratio of CHCl_3_: MeOH: citrate buffer). Extracts were subsequently vortexed for one minute and left at room temperature to separate over two hours before centrifugation for 10 min at 650 RCF. The supernatant was subsequently transferred to a CHCl_3_ rinsed glass tube. This step was repeated to ensure complete extraction of lipids from the peat pellet. Citrate buffer and chloroform (mixed at a 1:1 volume ratio) were left overnight to separate aqueous and organic phases. The layer of chloroform was transferred to a clean glass tube and blown dry under a stream of compressed N_2_ at room temperature^24^.

Lipids were separated using a silica solid phase extraction cartridge which was which was rinsed first with 15 mL methanol and 2.5 mL chloroform. The dry lipid extract was re-suspended in 0.5 mL chloroform and added to the column. Lipids were separated into neutral lipids (using chloroform), glycolipids (using acetone) and phospholipid fractions (using methanol). The PLFA fraction was collected and evaporated under a stream of compressed N_2_ in a heating block at 36 °C^37^.

PLFA fraction samples were re-suspended in 1 mL MeOH: toluene (1:1 volume ratio) and trans-esterified to fatty acid methy esthers (FAMEs), using 1 mL 0.2 M KOH dissolved in methanol. For liquid extraction, 2 mL of hexane: chloroform (4:1 volume ratio), 0.3 mL acetic acid (1.0 M), and 2 mL ultrapure water were added. Two internal standards (C13 and C19) were added to the samples before evaporating FAMEs under a stream of compressed N_2_.Samples were resuspended in hexane prior to GC analysis. PLFAs were identified and quantified using gas-chromatography^37^.

Individual PLFAs were identified using gas chromatography mass spectrometry (GC-MS) using an Agilent Technologies 5973 Mass Selective Detector (electron impact ionisation 70Ev, scan mode) coupled to an Agilent Technologies 6890 GC fitted with a 50 m × 0.32 mm i.d. × 0.25 μm CP-Sil 5CB fused silica capillary column. The temperature program was as follows: 50 °C (5) – 150 @ 10 °C min^− 1^ – 270 @ 3 °C min^− 1^ – 320 @ 20 °C min^− 1^.

δ^13^C values of individual PLFAs were analysed using gas chromatography-combustion-isotope ratio mass spectrometry (GC-C-IRMS). Compounds were first separated using an Agilent Technologies 6890 series gas chromatograph (splitless mode; 50 m x 0.32 mm x 0.2 μm CP-SIL 5CB column). The temperature was held isothermally at 50 °C for 5 min and then ramped from 50 to 150 °C at 10 °C min^− 1^; to 270 at 3 °C min^− 1^; to 340 at 20 °C (with a 5 min). He_2_ was used as the carrier gas. The GC effluent was diverted via a heart split union to a ceramic combustion furnace (650 mm × 0.3 mm i.d.) which was packed with a copper oxide/platinum/nichrome catalyst wire which was heated to 940 °C. Water was removed from the combustion products by passing the effluent through a nafion membrane, before the CO_2_ entering the isotope ratio mass spectrometer (IRMS) (Isoprime Ltd). PLFA δ^13^C values were corrected for the additional carbon atom introduced during methylation, using a correction factor obtained by CF-EA-IRMS measurement on the derivatising methanol and application of the mass balance Eq. 4^4^, where N_PLFA_ is the number of carbon atoms in the PLFA molecule, ^13^CFAME is the δ^13^C values of the methylated PLFA, and ^13^CMeOH is the δ^13^C value of the methanol used for methylation (-37‰):

2 $${\delta ^{{\text{13}}}}{{\text{C}}_{{\text{PLFA~}}}}=\frac{{\left( {{{\text{N}}_{{\text{PLFA}}}}+{\text{1}}} \right) \times {\delta ^{{\text{13}}}}{{\text{C}}_{{\text{FAME~}}}} - {\text{~}}{\delta ^{{\text{13}}}}{{\text{C}}_{{\text{MeOH~}}}}}}{{{{\text{N}}_{{\text{PLFA}}}}}}$$

Standard PLFA nomenclature (A: BωC) was used, where A refers to the total number of carbon atoms, B refers to the number double bonds, and C refers to the location of double carbon bonds. ‘A’, ‘i’, ‘cy’, and ‘me’ refer to anteiso-, iso-, cyclopropane and methyl groups, respectively^45^. C15:0i, C15:0a, C16:0i, C17:0i and C17:0a PLFA biomarkers were assigned to Gram positive bacteria. C16:1ω7, C17:0, C18:1ω7, and 7,8Cy-C19:0 were assigned to Gram negative bacteria. C18:2ω6c and C18:1ω9c were assigned to fungal biomarkers. C14:0, C15:0, C16:1ω6, C16:0, C17:1ω8, 10Me-C16:0, C17:1, C18:0, and 10Me-C18:0 biomarkers were left unclassified due to a lack of specificity to any microbial group^37^.

### Peat biogeochemical properties

Moisture content was determined by through the mass of water lost from 10 g wet weight peat oven dried at 105 °C for 24 h. Organic matter content was determined as the mass lost after ignition for 7 h at 550 °C. pH, conductivity and redox potential in each pot were determined using a 1:5 ratio of peat to deionized water. Total carbon (C) and total nitrogen (N) were quantified from 0.2 g of dry, homogenised peat and combusted using a total element analyser (Flash EA 1112, CE Instruments, Wigan, UK).

### Isotopic calculations

Natural abundances of ^13^C are typically expressed as δ^13^C (‰), which describes the ratios (R) of ^13^C and ^12^C relative to the standard. δ^13^C values were calculated as:

3 $${\delta ^{{\text{13}}}}{\text{C~}}=\frac{{{{\text{R}}_{{\text{sample}}}} - {{\text{R}}_{{\text{standard}}}}}}{{{{\text{R}}_{{\text{standard}}}}}} \times {\text{1000}}$$

Atom %, the absolute number of atoms of a given isotope in 100 atoms of an element^29^, was calculated in our pot experiment from labelled CH_4_ fluxes and PLFAs relative to pre-pulse measurements as:

4 $${\text{Atom}}\% =\frac{\left({100 \times AR\times }\left(\frac{{{(\delta}}^{{13}}\text{C}post{)}}{{1000}}{+\:1}\right)\right)}{\left(\text{1+AR}\times \left(\frac{{{(\delta}}^{{13}}\text{C}pre{)}}{{1000}}{+\:1}\right)\right)}$$

where AR equals the absolute ratio (0.0112372) of PDB standard material. Atom % was subsequently used to calculate ^13^C enrichment of CH_4_ fluxes relative to unlabelled natural abundance control plants of the same species (Table 1). ^13^C excess was calculated as:

5 $$\:{\:}^{13}C\:atom\:\left(\%\:excess\right)\:=\:atom\:{\%}_{labelled}-atom\:{\%}_{natural\:abundance}\:$$

CH_4_ fluxes were calculated using the ideal gas law and assuming the linear accumulation of gases over time within the chamber. CH_4_ fluxes from labelled plants comprised both pre-existing natural abundance ^13^C as well as ^13^C derived from the pulse. Natural abundance ^13^C was quantified using measurements from prior to labelling and from unlabelled plants, which were comparable. Excess CH_4_ was calculated as CH_4_ post-labelling minus mean CH_4_ flux from unlabelled plants. The ^13^CH_4_ flux (ng) was calculated using atom % data and the net flux rate.

**Table 1:** δ^13^ natural abundance for palm and broadleaved trees for CH_4_ and PLFAs, and C4 added sugar.

Component	Natural abundance δ^13^C (‰)
CH_4_	− 65.4 ± 2.9
PLFAs	− 29.6 ± 1.0
C4 sugar	− 12.11 ± 0.009

Means ± 1 SE.

### Statistical analyses

Differences in CH_4_ fluxes between paired girdled and control trees were assessed using a mixed effects model. CH_4_ fluxes were transformed using the box-cox transformation (CH_4_ ^− 0.2^). Differences in δ^13^C, atom % ^13^CH_4_ fluxes, PLFA abundances and PLFA enrichment were also assessed using a linear mixed effects model. Statistical models of CH_4_ fluxes included plant types and sampling day. Excess ^13^CH_4_ fluxes were log-transformed Significance was assessed *p* ≤ 0.05. All statistical analyses were conducted in GenStat (v17.1).

## Results

### Tree girdling

**Fig. 1:**
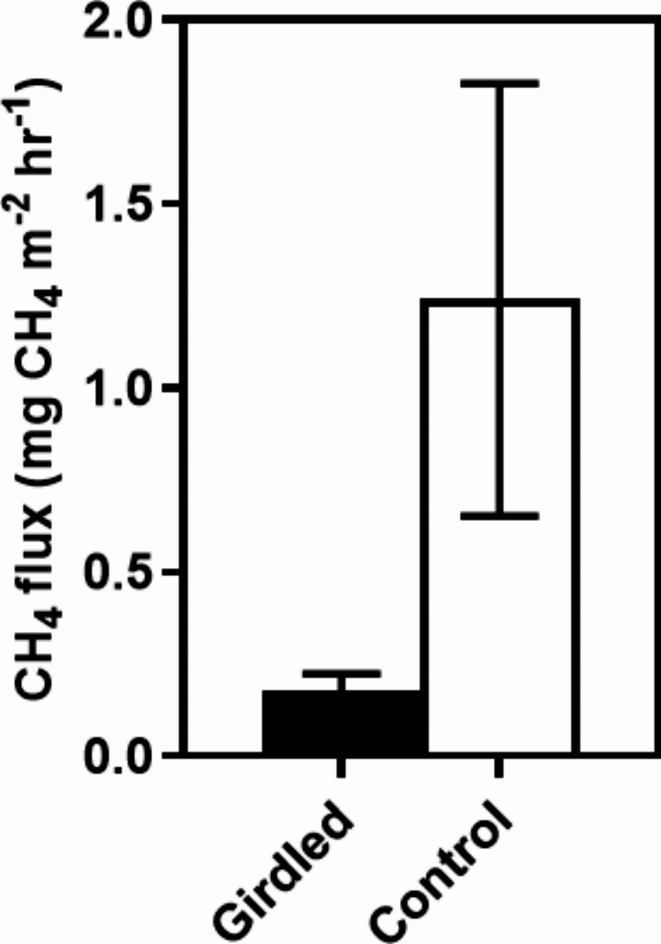
Mean CH_4_ fluxes measured 1- and 14-days post-girdling versus the non-girdled controls. Means ± 1 SE (*n* = 5).

Prior to girdling, the peat ranged from being a small source to a small sink of CH_4_. Immediately following girdling, there were no significant differences in CH_4_ fluxes between girdled trees and the controls (*p* = 0.934; Fig. 1). However, by 14 days post-girdling, CH_4_ fluxes adjacent to girdled trees were significantly lower than the controls (*p* = 0.047). Mean CH_4_ fluxes were 67 ± 14% lower from girdled trees, suggesting a substantial contribution of root inputs to peat surface CH_4_ fluxes.

### Natural abundance labelling

**Fig. 2:**
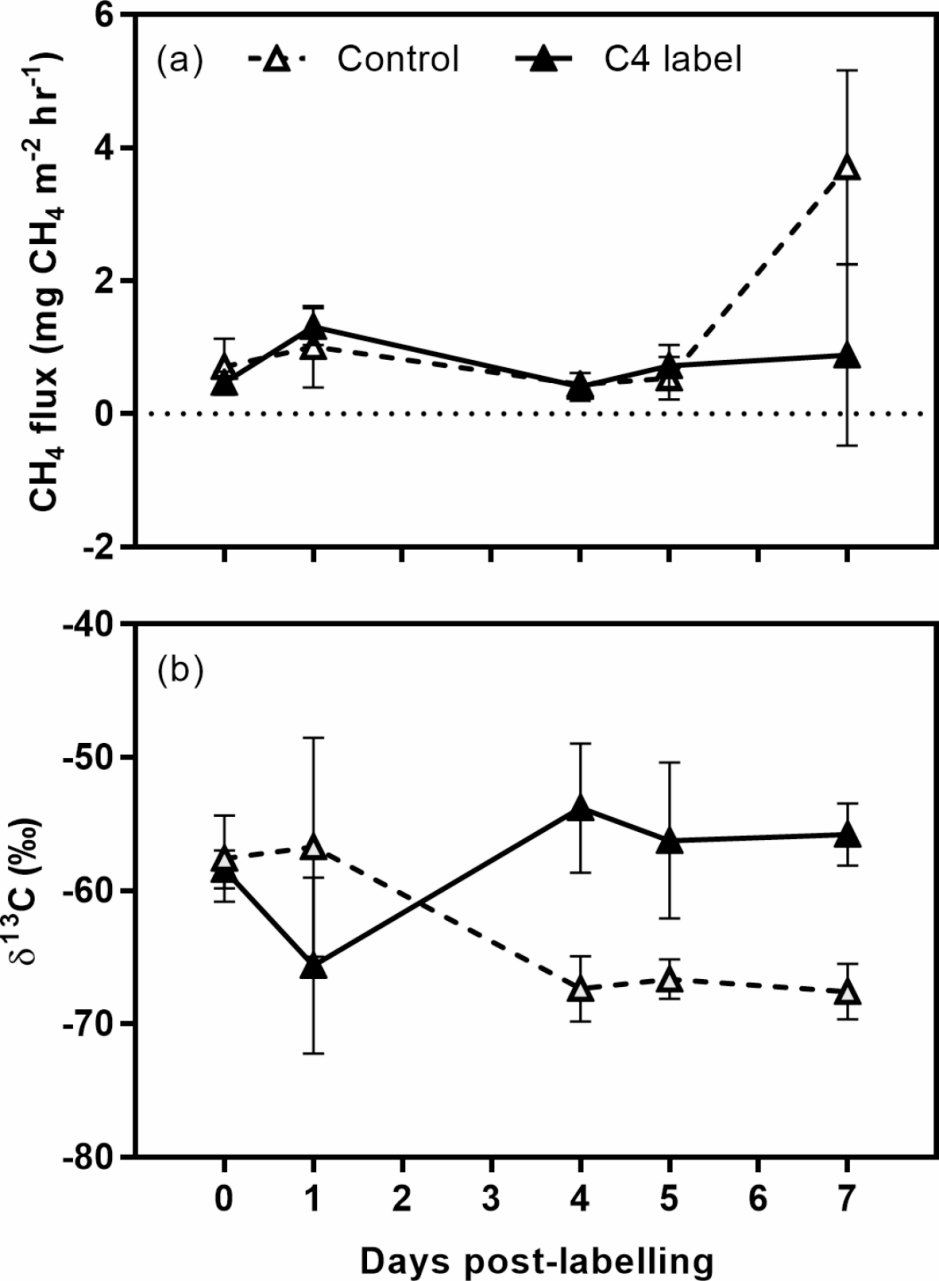
(**a**) Peat surface CH_4_ flux and (**b**) natural abundance labelling. Means ± 1 SE (labelled *n* = 4, control *n* = 3).

CH_4_ fluxes indicated that the peat was a consistent source of CH_4_ (Fig. 2a), with peak fluxes of 3.7 mg CH_4_ m^− 2^ hr^− 1^ and mean fluxes of 1.02 ± 0.31 mg CH_4_ m^− 2^ hr^− 1^. Addition of the natural abundance label resulted in a significant increase in the δ^13^C signature of CH_4_ compared to the controls (F_1,30_ = 7.44, *p* = 0.011), with the response observable within four days of labelling (Fig. 2b).

### ^13^CO_2_ labelling

**Fig. 3:**
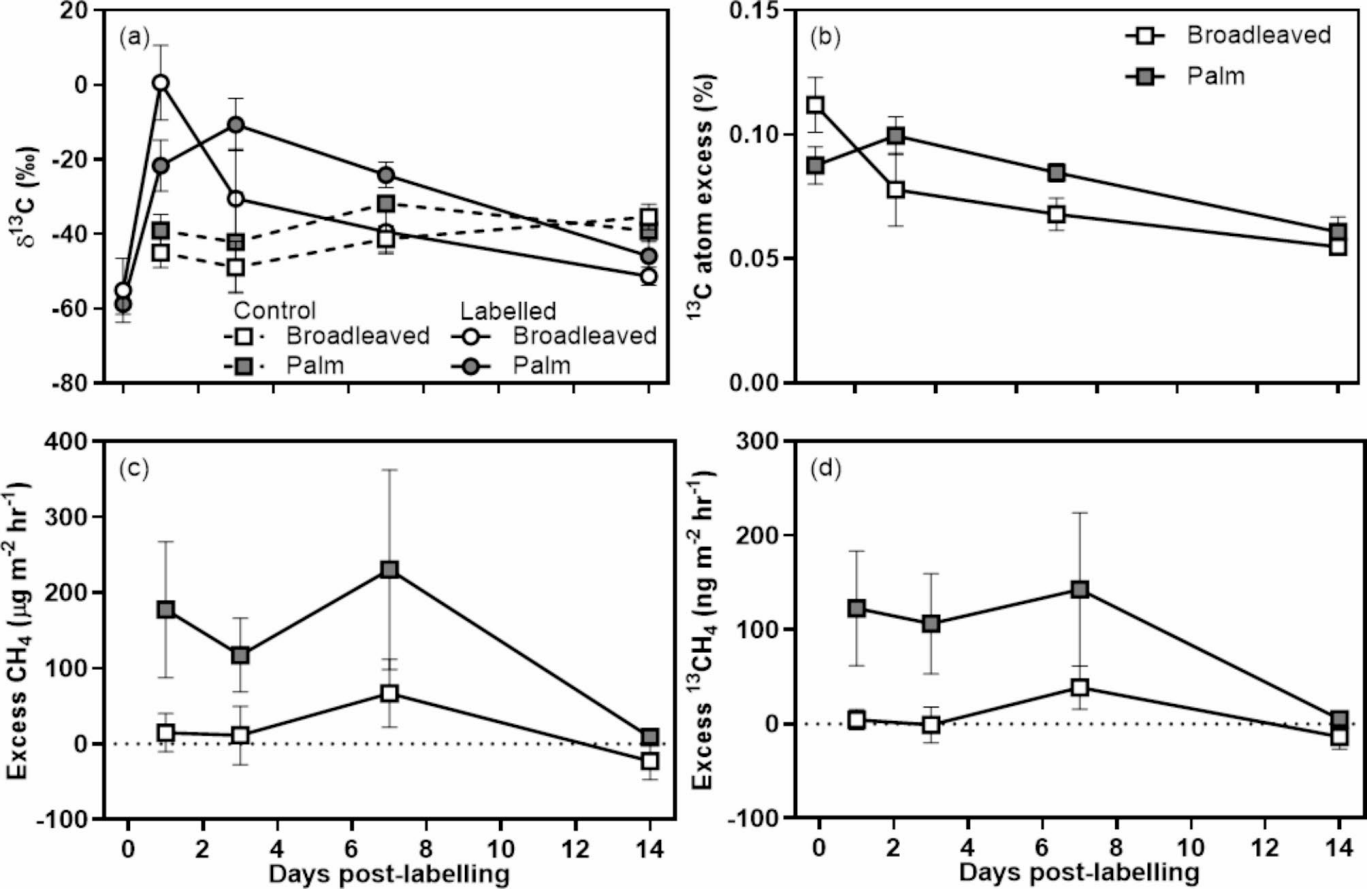
(**a**) δ^13^C of emitted CH_4_, (**b**) Atom excess ^13^CH_4_, (**c**) excess CH4 fluxes, (**d**) excess ^13^CH_4_ for broadleaved evergreen and palm plant types. Means ± 1 SE (broadleaved *n* = 5, palm *n* = 4).

CH_4_ emissions measured under both plant types showed significant enrichment 24 h following pulse-labelling (F_4_,_26.6_ = 12.9, *p* < 0.001, Fig. 3a,b), with ^13^C atom excess decreasing over time. There was no significant difference in ^13^C atom excess between plant types (F_1_,_7_ = 0.34, *p* = 0.58), but there was a significant interaction between plant types and number of days post-labelling (F_4_,_26.6_ = 2.81, *p* = 0.045), indicating differences in the rate of response between species.

Excess CH_4_ fluxes differed significantly between different plant types (F_1_,_7_ = 11.45, *p* = 0.01). Mean fluxes from palms were significantly larger than those of broadleaved evergreen saplings (Fig. 3c). Although fluxes were variable over time, and there was a large decline in fluxes 14 days post labelling (coinciding with a decline in ^13^C atom excess), there was no significant difference in excess ^13^CH_4_ fluxes over time (F_3_,_21_ = 1.63, *p* = 0.21). Moreover, as palm fluxes were consistently larger, the interaction term between plant type and days post-labelling was also not significant (F_1_,_21_ = 0.33, *p* = 0.80). Excess ^13^CH_4_ was significantly greater under palms (F_1_,_7_ = 22.62, *p* = 0.002, Fig. 3d). Between one and seven days post-labelling, the excess ^13^CH_4_ fluxes remained consistently above 100 ng m^− 2^ hr^− 1^, but by day 14 declined to almost zero, through a combination of declining ^13^C atom excess, and reduced net CH_4_ fluxes.

### PLFA and 13C-PLFA

**Fig. 4:**
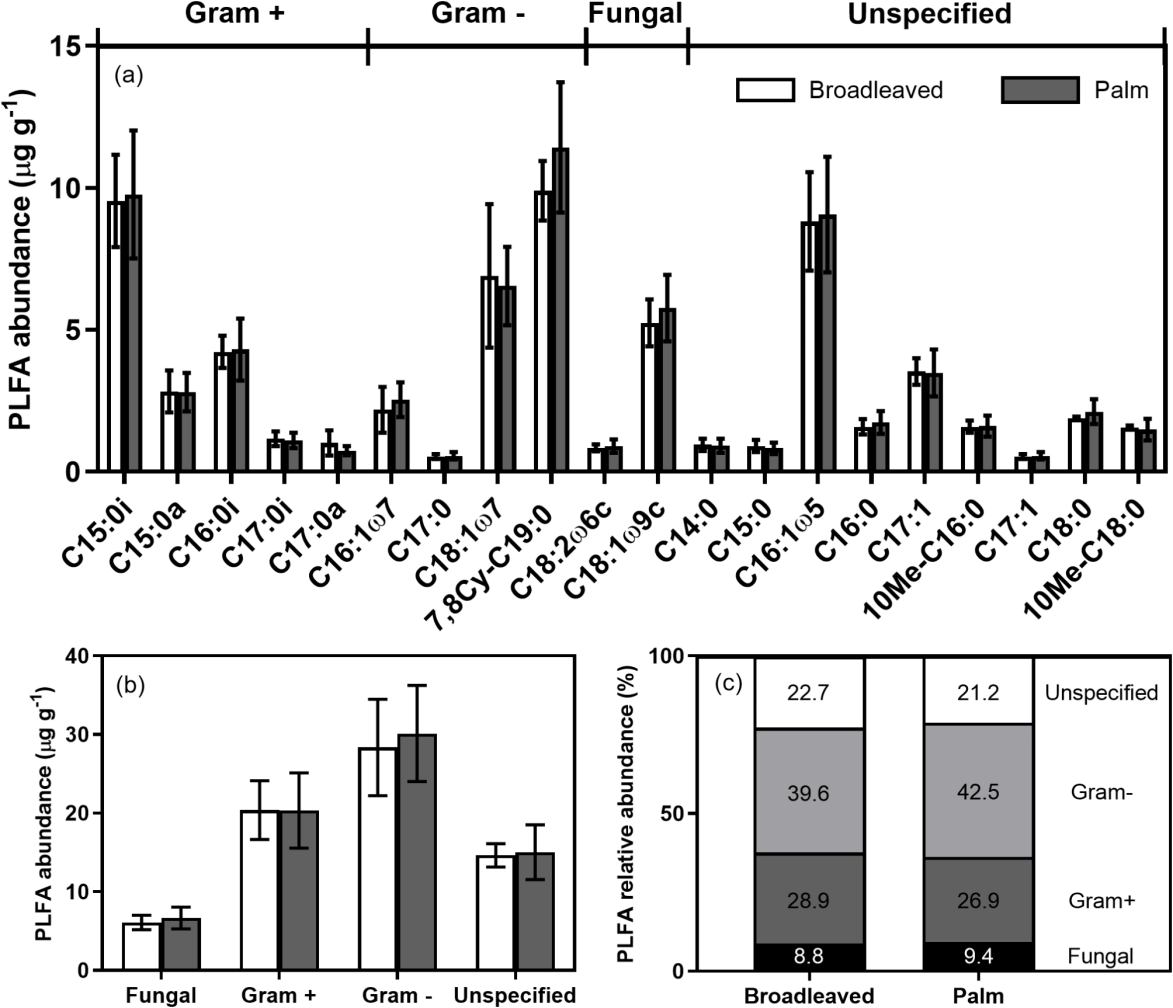
(**a**) Individual PLFA biomarker abundance, (**b**) PLFA abundance for fungal, Gram positive (G+), Gram negative (G-), and unspecified microbial groups, (**c**) Relative PLFA group abundance. Means ± 1 SE (*n* = 4 and 5).

There was no significant difference in total PLFA concentrations between broadleaved and palm plant types (F_1_,_7_ = 0.02, *p* = 0.90), and no difference between fungal (F_1_,_7_ = 0.11, *p* = 0.75), total bacterial (F_1_,_7_ = 0.01, *p* = 0.92), Gram positive (F_1_,_7_ = 0.0, *p* = 0.99) or Gram negative (F_1_,_7_ = 0.04, *p* = 0.85) PLFAs (Fig. 4b). As a percentage of total PLFAs, 8.8–9.4% of PLFAs were fungal, 26.9–28.9% were Gram positive, 39.6–42.5% were Gram negative, and 21.2–22.7% were non-specific (Fig. 4c). Fungi-bacteria ratios were consistent between plant types (0.13), as were ratios of Gram positive to Gram negative bacteria (0.64–0.73).

**Fig. 5:**
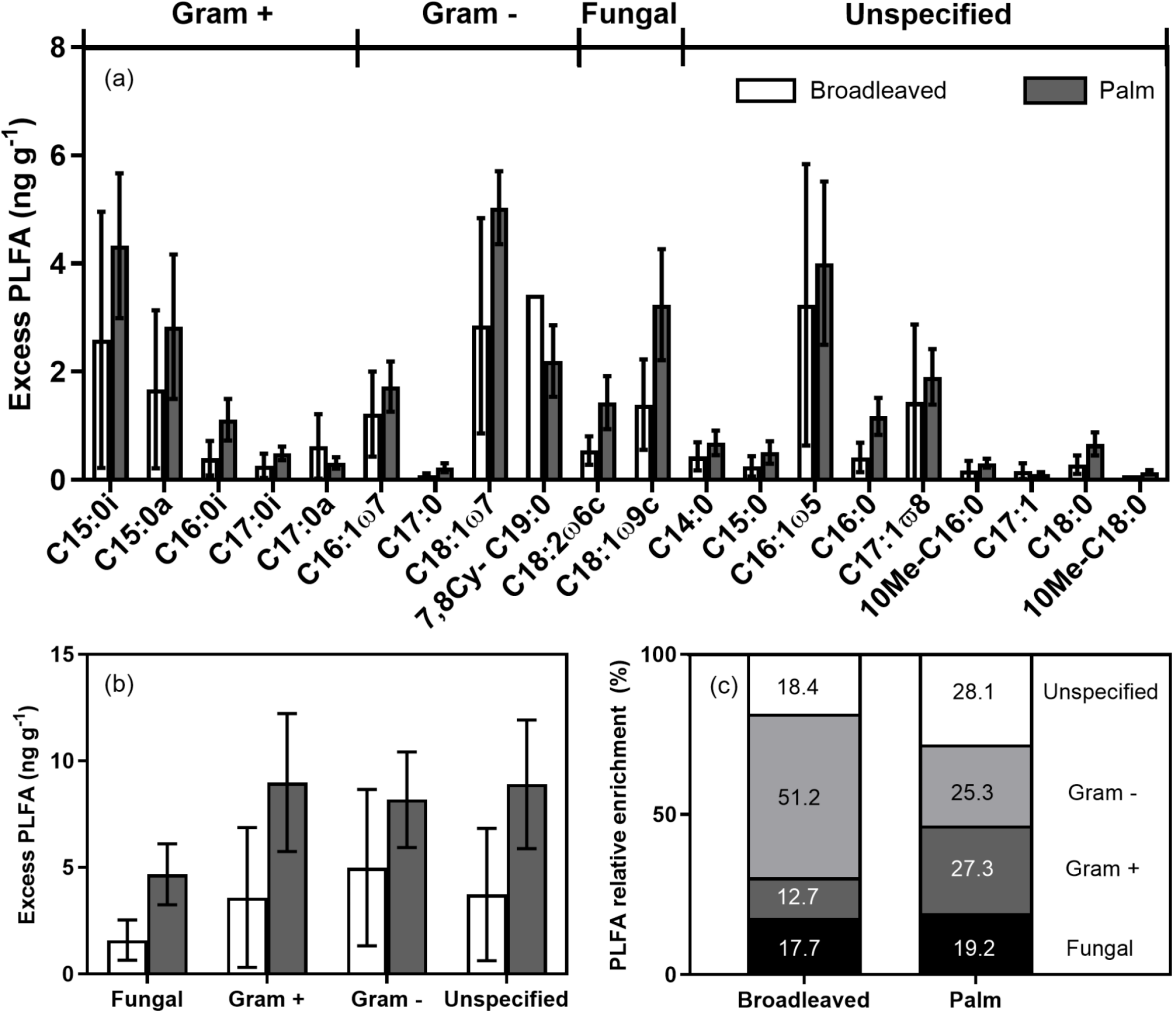
(**a**) Excess PLFA biomarker enrichment, (**b**) PLFA enrichment for fungal, Gram positive (G+), Gram negative (G-), and unspecified microbial groups, (**c**) Relative PLFA group enrichment. Means ± 1 SE (*n* = 4 and 5).

There was widespread enrichment of PLFA biomarkers seven days post labelling (Fig. 5a). There was, however, no significant difference in excess total (F_1_,_7_ = 1.35, *p* = 0.28), bacterial (F_1_,_7_ = 1.02, *p* = 0.35), fungal (F_1_,_7_ = 2.87, *p* = 0.13), Gram positive (F_1_,_7_ = 1.34, *p* = 0.29), or Gram negative (F_1_,_7_ = 0.6, *p* = 0.46) PLFAs (Fig. 5b). There were, however, significant differences in the percentage enrichment of both Gram positive and Gram negative PLFAs relative to total PLFA enrichment (Fig. 5c). Gram positive PLFAs were significantly more enriched for the palm (F_1_,_7_ = 6.39, *p* = 0.04), whereas Gram negative PLFAs were more enriched under the broadleaved evergreen plants (F_1_,_7_ = 5.70, *p* = 0.048).

## Discussion

### Root carbon inputs drive CH4 fluxes

The results from our studies demonstrate that exudates of carbon compounds from roots contributed to methane production and emissions. The 67% decrease in CH_4_ fluxes following girdling demonstrates that plant root inputs contribute significantly to peat surface emissions. This high apparent proportion of root contribution to surface CH_4_ fluxes is supported by the approximately two-third contribution of roots to CO_2_ fluxes we have previously demonstrated at the site^46^. Girdling will also have driven a reduction in rhizosphere respiration (including root respiration) by reducing the flow of exudates to the roots and peat^21^. It may also have reduced oxygen consumption within the rooting zone therefore leading to favourable conditions for methanotrophy although plant stem adaptations to supply oxygen to roots (lenticels) which are present for both *C. panamensis* and *S. globulifera* may have confounding effects^10^.

The significant enrichment following stem labelling in situ provides additional supporting evidence of the pathway, namely labile carbon transported in the plant vascular tissue contribute to CH_4_ emissions from tropical peatlands. The relatively high baseline values compared to post-labelling measurements most likely reflects natural environmental variability in the δ^13^C signature of CH_4_ arising from plant diurnal cycles, changes in water level, and temperature (Fig. 2b)^47^. The ^13^CO_2_ labelling results suggest that the contribution of root carbon inputs to CH_4_ fluxes varies between contrasting plant functional types, for example palms versus broadleaved evergreen trees, and that root inputs of carbon are derived from recent photosynthetically fixed carbon. We also demonstrate that a varied microbial community is responsible for utilising plant carbon inputs.

Previous studies investigating the role of labile carbon inputs on peatland CH_4_ dynamics have proposed a possible positive priming effect^48–50^, whereby the addition of an alternative carbon source (for example root exudates or plant litter) drives an increase in the microbial utilisation of organic matter^51^, although results are not always consistent between studies^8^. In our study we do not show any direct evidence of priming effects.,. These differences may be a consequence of the importance of root exudate component composition and concentration in regulating responses^9^. However, our results do demonstrate the close coupling of plant productivity and CH_4_ production. As a result, our findings have profound implications in assessing the potential response of peatland GHG fluxes to environmental change including alterations in temperature and increases in atmospheric CO_2_. Other studies have also highlighted the close correlation between plant productivity and wetland CH_4_ production^13,16^ and as a consequence any process which alters plant root inputs could significantly affect peat surface CH_4_ fluxes, possibly mediated through changes to both the composition and concentration of root exudate profiles^8,9^. These effects may be further exacerbated when combined with the high degree of temperature sensitivity of peat CH_4_ fluxes, and the projtected climate warming of tropical peatlands in the future^52–54^. Evidence that CH_4_ dynamics may respond to exogenous labile carbon input raises the possibility that, depending on the any changes in the relative allocation of labile vs. recalcitrant litter input from trees, may shift peatlands away from a current carbon input/output equilibria.

### The role of plant functional types

The isotopic enrichment of CH_4_ fluxes (Figs. 2a and 3) demonstrates a conclusive link between ongoing recently fixed carbon and methanogenic activity in tropical peatlands. In tropical wetland ecosystems, this link has previously only been demonstrated in rice paddy soils^55^. The response was relatively rapid, with changes in δ^13^C measured within 24 h of labelling in the case of saplings, and by four days following natural abundance labelling. The rapid response following ^13^CO_2_ labelling is most likely the result of a relatively short path length (all plants were less than 50 cm tall) which would allow rapid transfer of the label to the roots prior to exudation. Mean residence time is partially dependent on plant size and height, with full grown trees having longer residency in plant tissues^56^. There was no significant effect of girdling immediately following treatment, with effects detectable within 14 days. This likely reflects lags in the shutdown of delivery of metabolites, combined with the continued exudation of stored carbon in roots.

The significant interaction between plant type and number of days post-labelling suggests more rapid transfer of recently fixed carbon under broadleaved plants compared to palms, although the limited number of species investigated means that it is hard to ascribe any differences to a plant functional type effect rather than specific species differences. Mean residency times in leaf tissues are known to be variable between contrasting plant functional types, such as plants vs. shrubs^57^ and C4 vs. C3 plants^58^. Previously, it has been suggested that different plant functional types may respond differently to changing environmental conditions within chambers during labelling and sample collection, possibly resulting in differences in assimilation of ^13^CO_2_^29,59^. Moreover, palms and broadleaved trees are known to have distinct differences in vascular tissue between monocotyledonous and dicotyledonous plants, with monocotyledonous species (which includes palms) featuring vascular bundles scattered throughout the stem, compared to a distribution around the edges of the stem in dicotyledonous species^60^. These differences may, in part, account for observed differences in fluxes, as plant vascular tissue can be a significant conduit for gas transport from the soil to the atmosphere^61^. Further differences may be due to contrasting root exudate inputs, as root exudate composition and concentration is known to vary between plant species^8,9^.

### Microbial assimilation of 13C in tropical peatlands

PLFA biomarkers have been widely used to profile peat microbial community structure, as they are ubiquitous membrane spanning lipids found only in live cells and not microbial necromass^37,62^. Moreover, when combined with stable isotope labelling, ^13^C enrichment of biomarkers is a useful tool for assessing differences in microbial community function.

Total and specific PLFA biomarker abundance were consistent between microbial communities under both plant types. Both peat types are dominated by Gram positive and Gram negative bacteria consistent with measurements made in other forest soils in Panama^10,37,63–65^. Previous microbial community characterisations of peat from Changuinola have noted a dominance of Acidobacteria (a phylum of Gram negative bacteria)^43^, a finding also reported for other tropical^66^ and temperate peats^67^. Fungal abundance was low (8.8–9.4%), possibly due to the anoxic conditions arising from continual inundation by the water Tables^40,43,66^.

Enrichment of a range of PLFA biomarkers demonstrates that the ^13^C pulse can be utilised by a broad range of microorganisms, a finding that demonstrates that recent photosynthates are important drivers of microbial carbon dynamics in tropical peatlands as in other ecosystems^26,68,69^. Enrichment was most pronounced in C15:0i and C15:0a biomarkers for Gram positive bacteria, C18:1ω7 for Gram negative, and C18:1ω9c for fungi. The significant enrichment of Gram negative biomarkers in both peat types indicates an important role in utilising labile carbon inputs^70,71^. Gram negative biomarkers were strongly enriched relative to Gram positive biomarkers for the broadleaved plants but less so for the palm (Fig. 5), suggesting functional differences between microbial communities under contrasting vegetation. PLFA biomarker enrichment was, however, measured 14 days following labelling, and therefore some enrichment of microbial groups may be driven not by direct use of root exudates but through secondary consumption of dead microbial and root biomass^63^. Previously, Gram positive bacteria have been suggested as important in utilising more recalcitrant carbon^22,34^, as well as assimilating label derived from dead fungal or root biomass rather than from root exudates^72,73^. Fungal biomarkers were also significantly enriched indicating that, despite low abundance, fungi are also important in tropical peatland decomposition processes. Previously, it has been proposed that peatland microbial communities exhibit a certain degree of functional redundancy, whereby both fungal and bacterial communities are able to utilise a similar range of substrates and drive microbial production of CO_2_^74–76^.

It should be noted that as a technique, PLFA analysis does not detect methanogenic Archaea as they contain ether-linked rather than ester-linked lipids^77,78^. PLFA biomarkers for methanotrophs have, however, been previously reported. PLFAs comprising C14 and C16 generally dominate type I methanotrophs (generally assumed to dominate in low CH_4_ environments), whereas C18 fatty acids predominate in type II methanotrophs (which favour greater CH_4_ concentrations)^79,80^. Specific type I methanotroph biomarkers include, C16:1ω5, C16:1ω7, C16:1ω8c and C16:1ω11c, and C18:1ω7c C18:1ω8c for type II methanotrophs^80–83^. In this study both 18:1ω7c, and 16:1ω5 were identified and were present at similar abundances (Fig. 4a), and showed significant enrichment (Fig. 5a) potentially indicating the presence of both type I and type II methanotrophs using enriched CH_4_^84^.

### Conclusion

We have demonstrated that peat inputs of labile carbon from roots, derived from recent carbon fixation, make a significant contribution to peat CH_4_ production and surface CH_4_ emission, and that the extent of this contribution varies significantly between contrasting plant functional types. Despite similar microbial community structure between plant types, there were significant differences in isotopic enrichment of Gram positive and Gram negative populations, with the former showing increased enrichment in peat under broadleaved evergreen plants. These results are of particular importance in understanding microbial community function under contrasting vegetation types, owing to differences in the extent of the response of CH_4_ fluxes and microbial community enrichment. The intrinsic relationship between plant productivity and peat CH_4_ production, mediated through plant root inputs, have significant implications for the response of peatland CH_4_ fluxes to environmental change.

## Electronic supplementary material

Below is the link to the electronic supplementary material.

Supplementary Material 1

Supplementary Material 2

## Data Availability

All data generated or analysed during this study are included in this published article [and its supplementary information files].
