# Supplementary material for: Plant root carbon inputs drive methane production in tropical peatlands
Source: Sci Rep. 2025 Jan 25;15:3244. doi: 10.1038/s41598-025-87467-w (PMC11762785; doi:10.1038/s41598-025-87467-w)
Supplement: Supplementary file 1 — Supplementary Material 1 [file 41598_2025_87467_MOESM1_ESM.docx]

P**lant root carbon inputs drive methane production in tropical peatlands**

# Supplementary information:

## Plant and peat properties

Plants for ^13^CO_2_ pulse labelling were selected for their similar heights and DBH and as a result, aboveground and belowground biomass was consistent between plant types. Aboveground and belowground biomass were 4.14 g and 2.31 g for broadleaved evergreen plants, and 6.56 g and 3.86 g for palms, respectively (Supplementary table 1). Peat moisture and organic matter content were consistently high in peat under both plant types with means of 87.8% and 93.9%, and 86.0% and 84.6% under broadleaved evergreens and palms, respectively. Peats were acidic with low but highly variable conductivity within plant types, and were all weakly reducing (redox < 300 mV). Peats had high total carbon (35.1 – 46.1%) and nitrogen (2.0 – 2.6%), and consistent C:N ratios between plant types (17.2 – 17.6).

#### **Supplementary table 1**: Plant type and peat properties of broadleaved evergreen and palm plant types. Means ± 1 SE (n = 4 and 5).

| **Plant type** | **Broadleaved evergreen tree** | **Palm** |
| --- | --- | --- |
| Height (cm) | 36.4 ± 11.2 | 38.9 ± 8.2 |
| DBH (cm) | 0.33 ± 0.10 | 0.38 ± 0.13 |
| Aboveground biomass (g) | 4.1 ± 4.0 | 6.6 ± 3.6 |
| Belowground biomass (g) | 2.3 ± 1.4 | 3.9 ± 4.2 |
| Peat moisture (%) | 87.8 ± 2.7 | 86 ± 4.7 |
| Organic matter content (%) | 93.9 ± 2.2 | 84.5 ± 18.1 |
| pH | 4.1 ± 4.1 | 4.4 ± 0.3 |
| Conductivity (µS) | 128.9 ± 52.8 | 96.8 ± 16.1 |
| Redox (mV) | 288.4 ± 13.8 | 293.5 ± 7.2 |
| C (%) | 46.1 ± 7.2 | 35.1 ± 5.1 |
| N (%) | 2.6 ± 0.4 | 2.0 ± 0.3 |
| C:N | 17.6 ± 1.6 | 17.2 ± 0.9 |
